# Enhanced Arctic Amplification Began at the Mid-Brunhes Event ~400,000 years ago

**DOI:** 10.1038/s41598-017-13821-2

**Published:** 2017-11-03

**Authors:** T. M. Cronin, G. S. Dwyer, E. K. Caverly, J. Farmer, L. H. DeNinno, J. Rodriguez-Lazaro, L. Gemery

**Affiliations:** 1 Eastern Geology and Paleoclimate Science Center, MS 926 A US Geological Survey Reston, Virginia, 20192 USA; 2 Division of Earth and Ocean Sciences, Nicholas School of the Environment, Duke University, Durham, North Carolina USA; 3 Lamont-Doherty Earth Observatory, Palisades, New York, USA; 4 Present Address: Princeton University Department of Geosciences, Princeton, New Jersey, USA; 5 Dept. Estratigrafía y Paleontología, Facultad de Ciencia y Tecnología, Univ. País Vasco, UPV/EHU, Apartado 644, 48080 Bilbao, Spain

## Abstract

Arctic Ocean temperatures influence ecosystems, sea ice, species diversity, biogeochemical cycling, seafloor methane stability, deep-sea circulation, and CO_2_ cycling. Today’s Arctic Ocean and surrounding regions are undergoing climatic changes often attributed to “Arctic amplification” – that is, amplified warming in Arctic regions due to sea-ice loss and other processes, relative to global mean temperature. However, the long-term evolution of Arctic amplification is poorly constrained due to lack of continuous sediment proxy records of Arctic Ocean temperature, sea ice cover and circulation. Here we present reconstructions of Arctic Ocean intermediate depth water (AIW) temperatures and sea-ice cover spanning the last ~ 1.5 million years (Ma) of orbitally-paced glacial/interglacial cycles (GIC). Using Mg/Ca paleothermometry of the ostracode Krithe and sea-ice planktic and benthic indicator species, we suggest that the Mid-Brunhes Event (MBE), a major climate transition ~ 400–350 ka, involved fundamental changes in AIW temperature and sea-ice variability. Enhanced Arctic amplification at the MBE suggests a major climate threshold was reached at ~ 400 ka involving Atlantic Meridional Overturning Circulation (AMOC), inflowing warm Atlantic Layer water, ice sheet, sea-ice and ice-shelf feedbacks, and sensitivity to higher post-MBE interglacial CO_2_ concentrations.

## Introduction

Arctic amplification involves changes in albedo, ocean-atmosphere heat exchange, sea ice and other factors^1,2^ expected from climate model simulations due to rising greenhouse gas concentrations. An amplified climate response in the Arctic to past climate changes is consistent with paleoclimate evidence for cold temperatures during glacials^3^ and high temperatures during warm periods such as the early Holocene, Last Interglacial^4^ and the Late Pliocene^5^. To examine the development of Arctic amplification, this study examines the 1.5 million year history of intermediate depth ocean temperature, sea ice and marine productivity in the central Arctic Ocean using sediment core paleoceanographic records.

**Figure 1:**
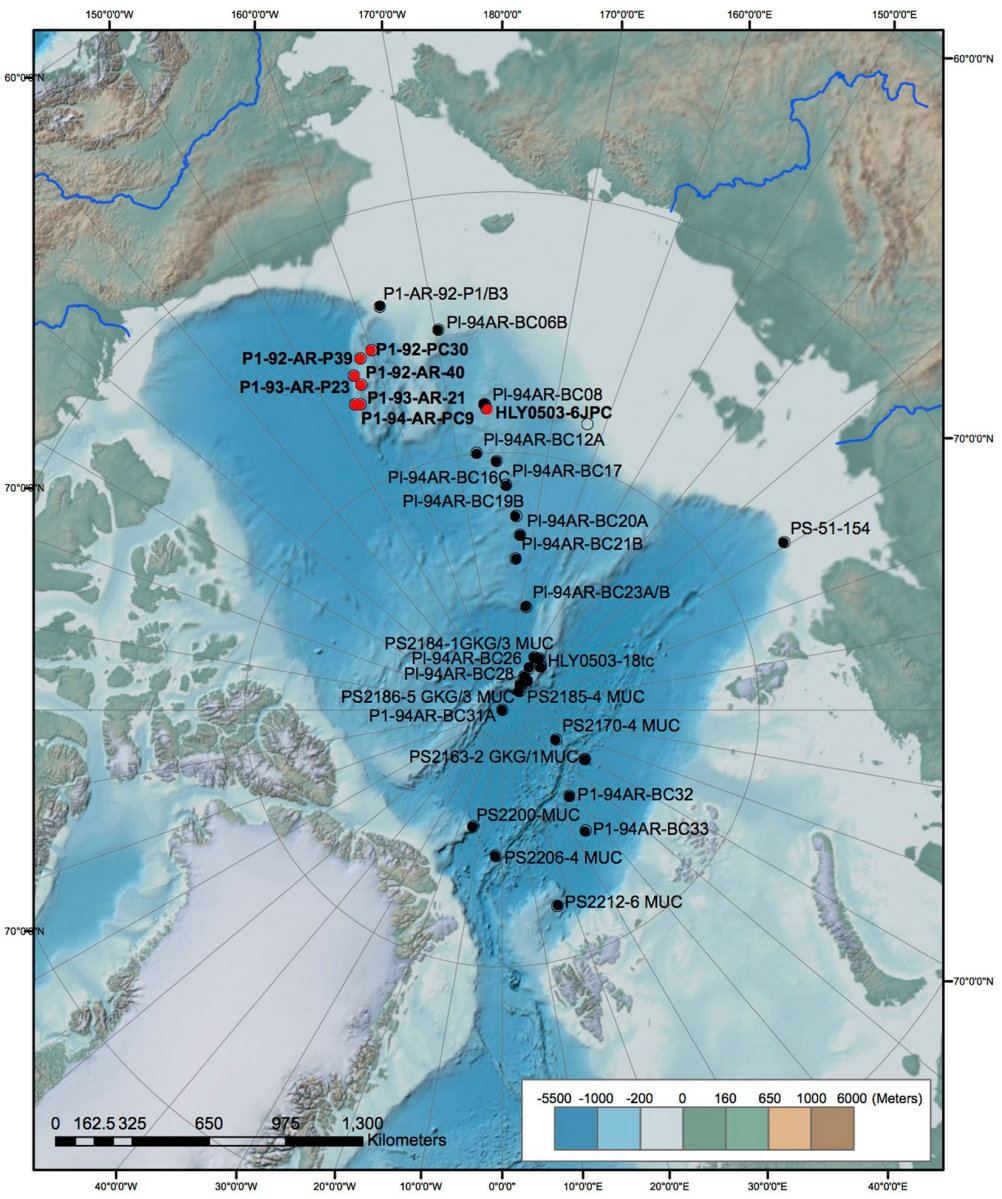
Map of Arctic Ocean showing Arctic core sites. Red circles are from current study covering the interval 50 ka–1.5 Ma, black circles are sites covering the last 50 ka from ref.^6^. See Appendix 1 for core site data. Basemap from National Oceanic and Atmospheric Administration National Centers for Environmental Information (NCEI); http://noaa.maps.arcgis.com/home/webmap/viewer.html?webmap=94f14eb0995e4bfc9d2439fc868345da ^30^.

Today’s central Arctic Ocean is characterized by a shallow halocline that separates the Polar Surface Layer and its perennial sea-ice from the warm Atlantic Water (200–500 m), which flows into central basins from the North Atlantic via the Fram Strait and Barents Sea. Variability in its strength has been linked to changes in the underlying AIW^6^ and the growth and decay of ice shelves^7^. Moreover, radionuclide proxy data indicate continuous deep-water exchange between the Arctic Ocean, the Nordic Seas and the North Atlantic Ocean over the last 35 ka^8^. To understand the changes in ocean circulation during the MBE, we reconstructed Arctic Ocean temperatures from benthic ostracode magnesium/calcium ratios (Mg/Ca), a tool for Arctic marine paleothermometry (Extended Data), and sea ice history from abundance patterns of ostracode species from 7 sediment cores from the western and central Arctic Ocean (Fig. 1). Both reconstructions mainly record interglacial and interstadial intervals, which are reflected in the abundances of calcareous microfossils^9^. Our new paleoceanographic records extend those of the last 50 ka^6,10^ back to ~1.5 Ma and reveal a fundamental shift in Arctic Ocean temperature, surface productivity and sea-ice cover variability at the MBE (Appendix 1). The age models for studied cores, which were based on radiocarbon ages in the upper parts of cores and orbitally tuned tiepoints based on microfossil abundance and the planktic foraminifera *Turborotalia egelida* (MIS 11) and the benthic species *Bulimina aculeata* (MIS 5c-5a) (ref.^9^). Data on these tiepoints and the estimated core depths for Marine Isotope Stratigraphy stages for each core are given in the Extended Data, which includes Supplementary Figure S1 and Supplementary Table 1.

## Results

**Figure 2:**
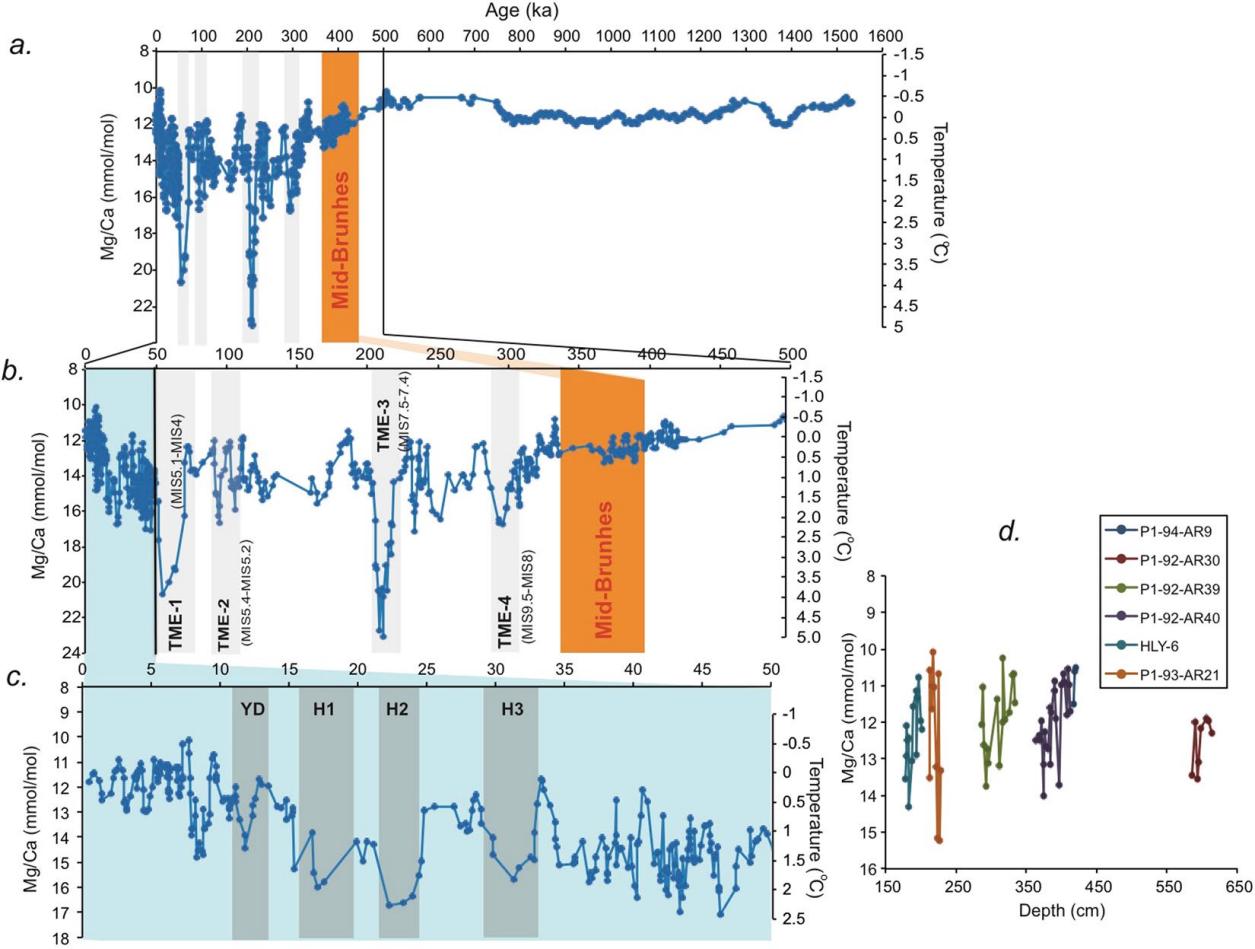
1.5 million-year Arctic Intermediate depth water temperature reconstructions from ostracode Mg/Ca. (**a**) Composite ostracode-Mg/Ca record from western Arctic Ocean sediment cores reflecting bottom water temperature changes over the last 1.5 Myr. Figure 2a is a 5-point moving average of a composite record from the current study of six new cores and the record from the last 50 kyr from ref.^6^. The record from 1.5 Ma to 0.6 Ma is exclusively from core P23 using two species of *Krithe*. (**b**) Expanded view of last 500 kyr record highlighting the four TME warming events. (**c**) Expanded view of last 50 kyr slightly revised from ref.^6^ with new data from additional cores, showing suborbital scale warming events associated with Heinrich Events and the Younger Dryas. (**d**) Detailed records of MIS 11 (~ 420–370 ka) from 5 cores plotted against core depth (cm).

Trends in ostracode Mg/Ca ratios and bottom intermediate water temperature (BWT) shown in Fig. 2a reveal a major transition beginning slowly, near 430 ka and increasing in amplitude after 350 ka, when Mg/Ca ratios rose from a pre-MBE level of 11–12 mmol/mol to post-MBE levels averaging around 14 mmol/mol. The Mg/Ca record from P1–93-AR-P23 (1.5 to 0.6 Ma, Fig. 2a, 953 m water depth) is based on measurements in two species of the benthic ostracode genus *Krithe*. The P23 stratigraphic record overlaps with those from other cores during the interval 560 to 360 ka. Converting the ostracode Mg/Ca ratios to temperature^11^ (Extended Data), the MBE shift equates to a rise in bottom temperatures in the AIW layers from near 0 °C to as much as 3–5 °C. Prior study of benthic foraminifera from core P23 first showed an Arctic Ocean dominated by seasonal sea ice transitioning to one dominated by abrupt sea-ice expansion during the mid-Pleistocene Transition and additional glacial expansion near the MBE^12^.

After 400 ka, the record is punctuated by 4 thermal maxima events (TME-1 through TME 4) where Mg/Ca values reached from ~15 to >22 mmol/mol (Fig. 2b). Importantly, TMEs seem to correspond to transitions from interglacial to glacial climates: TME-1 ~ 71–54 ka (MIS 5.1-MIS 4), TME-2 ~ 106–92 ka (MIS 5.4-MIS 5.2), TME-3 ~210–205 ka (MIS 7.4), TME-4 ~300–290 ka (MIS 9-MIS 8).

This pattern of brief AIW thermal maxima is supported by the hypothesis that rising AIW temperatures are related to thickening of the halocline and submergence of inflowing warm Atlantic Water, similar to the pattern documented over the last 50 kyr for the Younger Dryas (12 ka), and Heinrich events H1, H2 and H3 (17 ka, 24 ka, 32 ka, respectively; Fig. 2c)^6,13^.

Marine Isotope Stage 11 (MIS 11) centered at 400 ka, is an especially important interglacial period with sea level and high latitude temperatures higher than preindustrial and MIS 5.1^14^. MIS 11 sea-surface temperatures in the Arctic may have been as high as 8–10 °C^15^ but cooler than present in the Nordic Seas due to cool, low-salinity surface water^16^. Analysis of *Krithe* Mg/Ca ratios in 5 Arctic cores containing a well-preserved record of MIS 11 (Fig. 2d) show remarkably similar absolute values and consistent pattern of increasing in Mg/Ca, which presumably signifies the transition into the MIS 10 glacial period or MIS 11.2.

**Figure 3:**
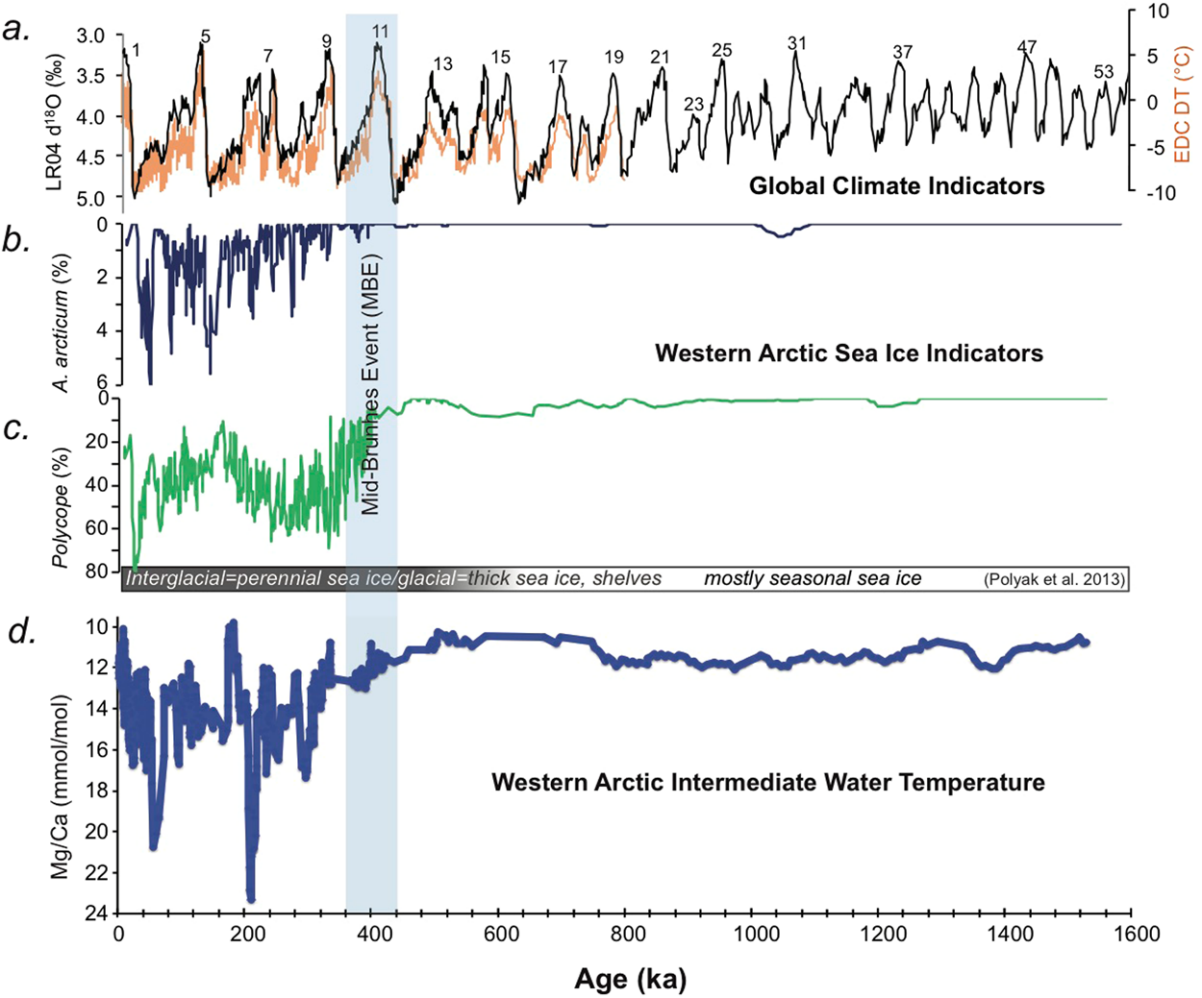
(**a**) Records of global climate. Global benthic foraminiferal oxygen isotope stack from ref.^17^ reflecting global continental ice volume and deep ocean temperature (black). Deuterium-based Antarctic temperature anomaly from EPICA Dome C ice core from ref.^18^ (orange). Blue-shaded area is the Mid-Brunhes Event. (**b**) Relative abundance of sea-ice related ostracode species *Acetabulastoma arcticum*, a parasitic species living on the sympagic epipelagic amphipod *Gammarus* in cores from the Western Arctic Ocean. Figure 3c is percent abundance of the genus *Polycope*, a benthic opportunistic genus signifying high local surface productivity in sea ice margin environments (**d**) Composite ostracode-Mg/Ca record from western Arctic Ocean sediment cores from Fig. 2 a reflecting bottom water temperatures the last 1.5 Myr. Continuous five-point moving average. TME-1 to TME-4 indicate periodic thermal maxima events during interglacial to glacial climatic transitions.

Arctic sea-ice cover and surface-ocean productivity are also closely tied to climatic and oceanographic change over various timescales^9^ and we used two ostracode groups as proxies for these parameters, *Acetabulastoma arcticum*, an obligatory parasite living on sea-ice dwelling amphipods, and *Polycope*, a benthic genus that is believed to be linked to pulses of high local surface productivity (Methods). Figure 3 compares the composite record of both taxa with the deep-sea benthic foraminiferal oxygen isotope curve^17^, the EPICA Dome C deuterium temperature curve^18^ (Fig. 3a) and the *Krithe* Mg/Ca record (Fig. 3c).

There is a brief *Polycope* spike during the MIS 16–15 cycle and a sustained increase in *Polycope* starting about 400 ka coincident with the rise in Mg/Ca ratios during the MIS 11 – MIS 10 transition. After the MBE, *Polycope* becomes a dominant taxon intermittently [mainly during stadials and interstadial] comprising 40 to >60% of total assemblages for much of the last 400 ka. Similarly, before ~350 ka, *A. arcticum* was virtually absent in the Arctic except for a few scattered occurrences, suggesting an absence of perennial sea ice, which is consistent with other microfaunal data (ref.^12^; Extended Data). *A. arcticum* occurs consistently starting about 350 ka often reaching percentages of 3–5% of the total assemblage, and 10–15% at individual core sites. We conclude from the three Arctic Ocean proxies that the MBE shift began about 400 ka with slightly higher AIW temperatures, then increasing AIW temperatures and surface productivity, culminating with the initiation of extensive sea ice and, during glacial maxima, probably large ice shelves by 350 ka.

## Discussion

**Figure 4:**
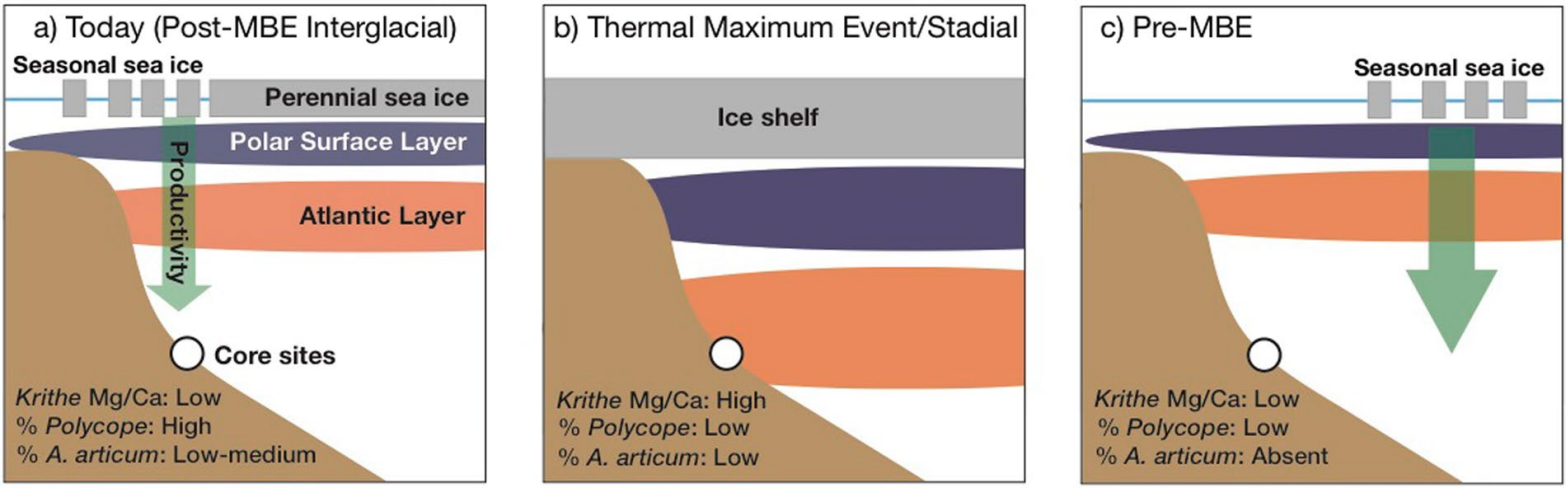
Conceptual view of Arctic paleoceanography regimes over the last 1.5 Myr. (**a**) Modern-day and post-MBE interglacial regime. Perennial sea-ice characterizes the central Arctic Ocean, with seasonal sea-ice at the ocean margins. Cold, low salinity Polar Surface Layer (purple) underlies sea-ice, and is separated from the warmer Atlantic Layer (orange) by a halocline. Productivity (green arrow) is locally enhanced at the ice margin. Faunal and geochemical records indicate low intermediate-depth temperatures, higher local productivity, and the presence of sea-ice. (**b**) TME/stadial regime. Extensive ice shelves and/or freshwater input drive a deepened Polar Surface Layer and Atlantic Layer, associated with higher intermediate-depth temperatures, low local productivity, and low sea-ice in our records. (**c**) Pre-MBE interglacial conditions. Sea-ice is likely absent outside of the Central Arctic, with higher productivity shifted to the area of seasonal sea-ice. Our records indicate lower intermediate-depth temperatures, low local productivity, and absent sea-ice.

Figure 4 provides a conceptual view of Arctic paleoceanography regimes over the last 1.5 Myr, which helps explain the abrupt shift in Arctic climate at the MBE, post-MBE thermal maxima events, Atlantic Water and underlying AIW and, more broadly, the evolution of Arctic cryosphere and climate. Figure 4a shows the modern regime with perennial sea ice in the central Arctic and seasonal sea ice along the margins. Examining the last 50 kyr, AIW temperature maxima were centered on the Younger Dryas (12 ka), and Heinrich events H1, H2 and H3 (17 ka, 24 ka, 32 ka, respectively, Figs. 2c,4b)^6^. These appear to be related to collapse and renewal of AMOC during Heinrich Events in the North Atlantic^19,20^.

Prior to 50 ka, the largest Arctic TME-3 around 210–205 ka, occurred during a strong stadial within MIS 7 (MIS 7.4) when sea level^21^, Antarctic temperature^17^ and atmospheric CO_2_ concentrations^22^ all fell sharply. TME-4 and TME-2 occurred at 300 to 290 ka, during the MIS 9-MIS 8 transition, and 106 to 92 ka, during MIS 5.4-MIS 5.2, respectively. Between 390 and 365 ka, there was a small but consistent increase in AIW temperature during the aforementioned MIS 11-to-MIS 10 transition. Although the precise ages of thermal maxima in AIW are uncertain due to low sedimentation rates, all TMEs, including those of the last 50 kyr, seem to occur during periods of climatic transition between interglacial and glacial periods.

It has been known for some time that the largest manifestations of climatic transition at the MBE were interglacial CO_2_ levels and deep-sea temperature^23^. Most other continuous proxy records spanning the MBE (see Extended Data)–deep-sea temperature, ice volume, sea level, global sea-surface temperature, East Asian Monsoon strength–show progressive change in the amplitude of orbital cycles through the MBE interval. In contrast, our data suggest that the Arctic Ocean (Fig. 4c), specifically sea ice [and perhaps also ice shelves], productivity and intermediate water temperature, shifted over several tens of thousands of years beginning ~400 ka. The AIW temperature shift at the MBE is, in turn, likely a reflection of changes in the strength of inflowing Atlantic water and AMOC.

The occurrence (absence) of TMEs after (prior to) ~ 400 ka reflects an enhanced sensitivity of Arctic climate, cryosphere and ocean circulation to relatively small differences in external forcing from insolation^18^ and higher post-MBE CO_2_ concentrations during the last five interglacial periods (280 ppmv post-MBE versus 240 ppmv pre-MBE)^23,24^. One factor that may explain this shift is that post-MBE northern hemisphere ice sheets and Arctic Ocean ice shelves were larger than those prior to the MBE. Growing evidence shows that the Arctic Ocean has been partially covered with thick (up to 1-km) ice shelves and sea-ice cover during recent glacial maxima (Extended Data).

If as our results suggest, the 40-ppmv higher post-MBE interglacial CO_2_ concentrations produced conditions conducive to large-scale ocean-ice changes within the Arctic Ocean, there may be implications for the global carbon cycle. For example, Bouttes *et al*.^25^ conducted carbon-cycle modeling of the MBE finding the magnitude of the pre and post-MBE CO_2_ difference cannot be accounted for by insolation, glacial ice sheet extent, or terrestrial carbon storage. Thus the Arctic Ocean may have contributed to the MBE CO_2_ change via temperature and sea-ice changes discussed above. We speculate that the Arctic Ocean system would act as a relative carbon sink during warm periods and a carbon source during cool periods. During pre-MBE interglacials, there would be greater CO_2_ and organic carbon storage in the Arctic Ocean Arctic Ocean and sediments, respectively, due in part to colder AIW temperatures and higher integrated surface Arctic productivity due to less perennial sea ice. In contrast, during post-MBE interglacial periods, warmer AIW temperatures and lower integrated surface Arctic productivity and increased sea ice would result in less CO_2_ and organic carbon production/storage in Arctic Ocean. Although the Arctic Ocean’s small size limits the possible magnitude of carbon storage, this differential Arctic Ocean carbon storage during interglacials would nonetheless complement Southern Ocean changes in deep ventilation^26^ and polar front position^27^ proposed to account for higher post-MBE interglacial CO_2_ concentrations. It is unclear why *Polycope* spp. and associated sea-ice margin species were extremely rare in the pre-MBE Arctic but it is likely due to the very different summer sea-ice conditions and competition with the diverse pre-MBE ostracodes (Extended data).

Southern Hemisphere ocean-atmosphere-sea ice processes are critical for understanding the MBE, specifically the idea that there is a bipolar seesaw operating between Northern and Southern Hemispheres on millennial timescales explaining warmer interglacial conditions in the Southern Hemisphere. Barker *et al*. (2011)^28^ demonstrated that abrupt millennial-scale AMOC variability characterized the last 800 ka, albeit without the large amplitude shift seen in our Arctic records. Holden *et al*.^29^ proposed a role for decreased stability of the West Antarctic Ice Sheet following the MBE, leading to AMOC slowdown during deglacials. Thus, it is possible that ice sheet/ice shelf instability characterized both hemispheres providing the necessary non-linear dynamics to explain large amplitude temperature events in the Arctic Ocean. However, establishing details of the timing of post-MBE suborbital events – especially the relationship between bottom temperature, sea ice and productivity during stadial and interstadial periods - requires better sediment core resolution in the Arctic. Nonetheless, the large shift in Arctic land ice, ice shelves and sea ice at the MBE, suggests an amplification of Arctic climate sensitivity related to higher interglacial CO_2_ concentrations and associated feedbacks involving ice shelves and ice sheets, Heinrich-like events, AMOC-forced Arctic Ocean temperature oscillations, and deeper submergence of Atlantic water in the central Arctic Basin.

All data presented in this manuscript have been deposited in the National Climatic Data Center (NCDC) (https://www.ncdc.noaa.gov/data-access/paleoclimatology-data).

## Electronic supplementary material

Supplementary Material 2 Appendix

Supplementary Material-Methods
