# Supplementary material for: Enhanced Arctic Amplification Began at the Mid-Brunhes Event ~400,000 years ago
Source: Sci Rep. 2017 Nov 3;7:14475. doi: 10.1038/s41598-017-13821-2 (PMC5670171; doi:10.1038/s41598-017-13821-2)
Supplement: Supplementary file 2 — Supplementary Material-Methods [file 41598_2017_13821_MOESM2_ESM.pdf]

Enhanced Arctic Amplification Began at the Mid-Brunhes Event ~400,000 years ago

T. M. Cronin[1]*G. S. Dwyer[2]*, E. K. Caverly[1], J. Farmer[3], L. H. DeNinno[1], J. Rodriguez-Lazaro[4] L. Gemery[1]

Online Content: Methods, **Extended Data** and **Appendix**.

Methods

Seven piston and gravity cores from the Northwind Ridge and Mendeleev Ridge in the Arctic Ocean (water depths 700-1470 meters) were used to extend the 50 kyr record from Cronin et al. (ref 6), which was based on 31 short box and multicores, back to 1.5 Ma. (Fig. 1). Chronology combined radiocarbon dating[10], bio- and lithostratigraphy[13, 31, 32], and orbital tuning[9] (Figure S1)

Adult ostracode shells were picked using fine brushes from the >150μm dry fraction and assigned VPI values. Three species of *Krithe* inhabit the Arctic: *K. hunti* (formerly called *K. glacialis*[33, 34]), which is the most common species in the last 400 kyr, *K. minima*, and *K. aquilonia*, which inhabits the pre-Brunhes interval[32].
Prior to Mg/Ca analysis, adult specimens of *Krithe* were soaked in ~5% NaOCl for 16-24 h to oxidize organic matter and remove adhering particles. Shells were triple-rinsed in deionized water, inspected under a light microscope for the remaining adhering particles, and twice more rinsed with deionized water under light sonication. Shells were then dissolved in 3-30mL of 0.05N nitric acid and the resulting aqueous solution analyzed for Mg, Sr, and Ca on a Fisons Instruments Spectraspan 7 direct current plasma atomic emission spectrometer (DCP) at Duke University using ultra-pure plasma-grade SPEX standard solutions. Analytical precision is approximately 2% based on replicate analysis of samples and standards.

Extended Data

Ostracode shell chemistry and paleothermometry

Previous studies indicate that *Krithe* shell Mg/Ca ratios are predominantly controlled by water temperature at the time of shell growth and that post-depositional diagenetic factors such as dissolution, have negligible impact on original shell Mg/Ca ratios[35, 36, 37, 38, 11]. Carbonate ion effects, which are thought to influence deep-sea benthic foraminifera Mg/Ca ratios[37, 38], do not appear to influence *Krithe* Mg/Ca, especially in Arctic Ocean sediments[11]. Finally, although dissolution can affect ostracode shells, dissolution has negligible impact on ostracode Mg/Ca ratios[38], but we nevertheless assess dissolution using a visual inspection index (VPI) that quantifies preservation state using VPI values of 1 [clear, translucent, pristine] to 7 [partially dissolved, chalky]. In our reconstruction we use well-preserved specimens (VPI – 1-5) that additionally show no signs of secondary calcite.

The Mg/Ca-temperature relationship for the main Arctic species of *Krithe*, *K. hunti*, is based on Arctic-Nordic Sea coretop material from 50 sites (50 to 3500m water depth, temperatures from -1.6° to 1°C) and is expressed in the equation BWT (ºC) = (0.438 x Mg/Ca_{Krithe}) – 5.14 ($r^2 = 0.5$). This calibration has a 1σ prediction error of ±1.0°C and a temperature sensitivity is ~2.3 mmol mol^{-1} $°C^{-1}$ [6,11]. This sensitivity is nearly double that of *Krithe* species from the North Atlantic (temperature range of 2° to 14°C)[37] but similar to that from Coral Sea *Krithe* Mg/Ca (temperatures 2° to 6°C). We point out one caveat that some Mg/Ca values fall outside the calibration range (up to 13 mmol/mol), so there is additional uncertainty with the actual BWTs they represent. *Krithe minima* is used in some Mg/Ca paleothermometry in the Arctic with a species vital effect correction based on consistent offset[11]. Although no modern calibration is available for the other Arctic species *K. aquilonia*, paired samples of *K. hunti* and *K. aquilonia* in the pre-Brunhes sediments from core P23 show indistinguishable Mg/Ca ratios for the two species.

In addition to temperature, we considered other potential factors that may have influenced Mg/Ca variability at the MBE. Although coretop Arctic *Krithe* Mg/Ca values show no carbonate ion sensitivity[11], we cannot totally rule out some effect

given the apparent magnitude of the biogeochemical changes in the Arctic Ocean across the MBE transition, as well as those over orbital and suborbital timescales. However, we note that our Mg/Ca results are inconsistent with the direction of a carbonate ion effect. If the Mg/Ca increase after the MBE were due to carbonate ion instead of temperature, this would require that AIW became enriched in carbonate ion after the MBE, and should be reflected in enhanced $CaCO_3$ preservation in both the Arctic and Nordic Seas. Though available records are limited, $CaCO_3$ preservation in the Nordic Seas does not support a preservation spike around the MBE[41].

Another possibility is that there was at certain times higher seawater Mg in the Arctic Ocean due to land-sea transport of dolomite, although it is difficult to imagine how this would affect bottom water dissolved ion chemistry, ostracode shell secretion, and the chemistry of molting fluids. This scenario is implausible for numerous reasons, including: (1) Ostracode Mg/Ca peaks in the record (TME's), including pre- and post-peak lower Mg/Ca values, occur stratigraphically outside dolomitic IRD layers, which are used as stratigraphic markers in the Arctic Ocean; (2) Dolomite is less soluble than calcite; thus water corrosive enough to dissolve dolomite and release Mg would likely dissolve all calcitic ostracode shells leaving sediment barren of ostracode shells; (3) Dissolution of dolomite would release Mg and Ca in 1:1 proportion thereby decreasing the seawater Mg/Ca ratio (~5:1), which in turn should actually lead to lower Mg/Ca ratios in ostracode shells, not higher.

Ostracode ecology and indicator species

Two indicator ostracode groups are used to reconstruct Arctic Ocean sea ice and productivity. The species *Acetabulastoma arcticum* has been used as a sea-ice proxy because it is a parasitic species living in the epipelagic, sea-ice dwelling amphipod *Gammarus* and its shells are commonly preserved in Arctic sediments[42]. The benthic genus *Polycope,* an opportunistic group that signifies high local surface ocean productivity[41], often reaches 80% of total assemblages in late Quaternary

sediments[10]. Prior study of *A. arcticum* and *Polycope* from many box and multicores shows large variability related to surface sea ice and productivity throughout most of the central Arctic[10]. These two ostracode taxa were studied in core HLY0503-6 from the Mendeleev Ridge indicating a sharp increase in both sea-ice and local productivity proxies between 400 and 350 ka[15, 32]. The near absence of *Polycope* spp. and associated species in pre-MBE sediments likely reflects its migration into the Arctic Ocean only when appropriate habitat, with local and pulsed surface productivity providing surface-to-bottom food for opportunistic genus. In addition, the sustained lack of summer sea ice and competition with a more diverse benthic ostracode fauna were likely factors influencing the pre-MBE benthic assemblages[32]. A major environmental change near the MBE is also indicated by benthic foraminiferal assemblages[13].

Revisionist views on Arctic Ice Cover during Glacials

There is growing evidence that the Arctic Ocean was at least partially covered with thick ice shelves up to 1-km thick and thicker-than-modern sea-ice cover during recent glacial periods. Evidence comes from geophysical and sediment records from the Hovgaard Ridge–Arctic Ocean[44], the Chukchi Borderland (including the Northwind Ridge and Chukchi Plateau[45, 46, 47], the Beaufort Sea[48] and East Siberian Sea[49] margins, the Lomonosov Ridge, the Arlis Plateau and the slope off the Herald Canyon, E. Siberian Sea[50, 51, 7]. Age estimates vary but include the last few major glacial maxima including MIS 2, 4, and 6. Laurentide Ice Sheet ice streams also exhibited complex behavior during periods of deglaciation[52], which may have contributed to large ice discharges during Heinrich Events.

Continuous Mid-Brunhes Records

Previously published continuous records across the MBE include deep-sea temperature[53], ice volume derived from deep-sea $\delta^{18}O$[17], sea level[54], global sea-surface temperature[55], atmospheric CO_2[22, 56], and East Asian Monsoon strength[57]. They generally show gradual, progressive change in the pattern of glacial-interglacial cycles with increasing amplitude over the last 5 cycles.

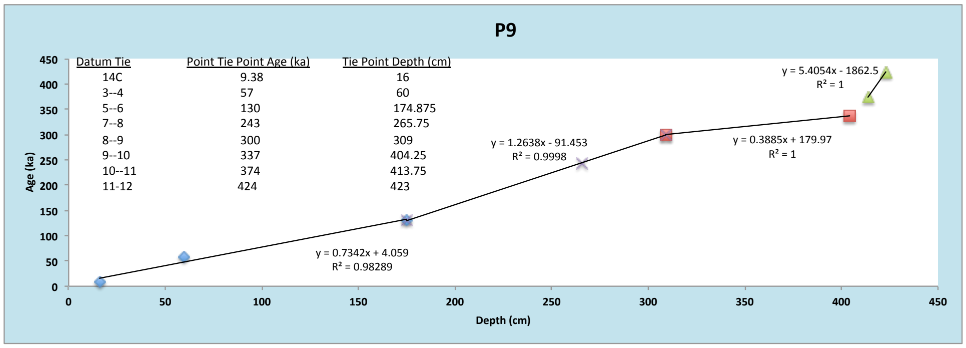
P9
Datum Tie
Point Tie Point Age (ka)
Tie Point Depth (cm)
14C 9.38 16
3--4 57 60
5--6 130 174.875
7--8 243 265.75
8--9 300 309
9--10 337 404.25
10--11 374 413.75
11-12 424 423
y = 5.4054x - 1862.5
R² = 1
y = 1.2638x - 91.453
R² = 0.9998
y = 0.3885x + 179.97
R² = 1
y = 0.7342x + 4.059
R² = 0.98289
Age (ka)
Depth (cm)
0 50 100 150 200 250 300 350 400 450

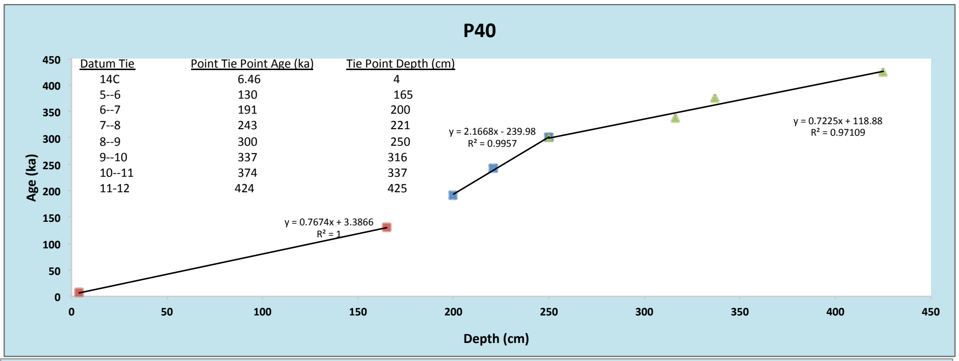
P40
Datum Tie
Point Tie Point Age (ka)
Tie Point Depth (cm)
14C 6.46 4
5--6 130 165
6--7 191 200
7--8 243 221
8--9 300 250
9--10 337 316
10--11 374 337
11-12 424 425
y = 2.1668x - 239.98
R² = 0.9957
y = 0.7225x + 118.88
R² = 0.97109
y = 0.7674x + 3.3866
R² = 1
Age (ka)
Depth (cm)
0 50 100 150 200 250 300 350 400 450

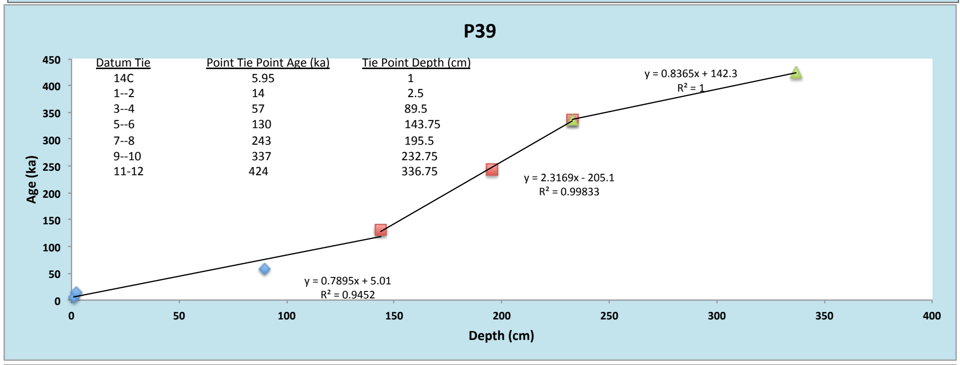
P39
Datum Tie
Point Tie Point Age (ka)
Tie Point Depth (cm)
14C 5.95 1
1--2 14 2.5
3--4 57 89.5
5--6 130 143.75
7--8 243 195.5
9--10 337 232.75
11-12 424 336.75
y = 0.8365x + 142.3
R² = 1
y = 2.3169x - 205.1
R² = 0.99833
y = 0.7895x + 5.01
R² = 0.9452
Age (ka)
Depth (cm)
0 50 100 150 200 250 300 350 400

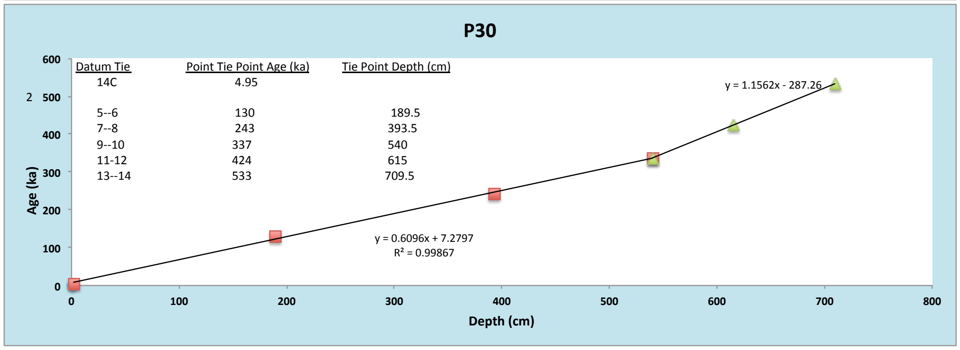
P30
Datum Tie
Point Tie Point Age (ka)
Tie Point Depth (cm)
14C 4.95
2
5--6 130 189.5
7--8 243 393.5
9--10 337 540
11-12 424 615
13--14 533 709.5
y = 1.1562x - 287.26
y = 0.6096x + 7.2797
R² = 0.99867
Age (ka)
Depth (cm)
0 100 200 300 400 500 600 700 800

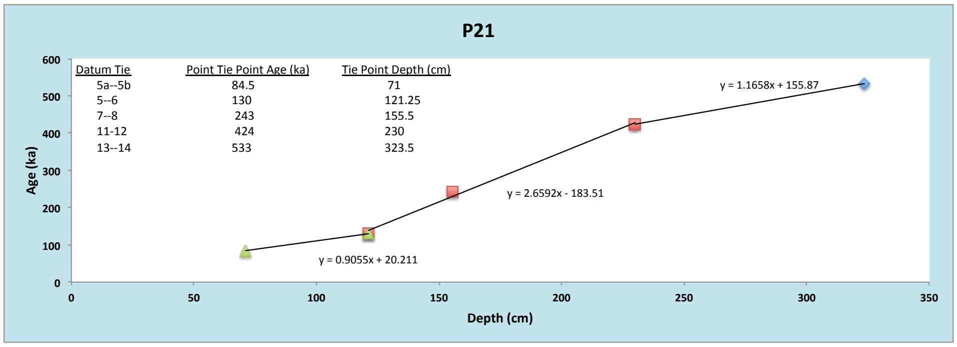
P21
Datum Tie
Point Tie Point Age (ka)
Tie Point Depth (cm)
5a--5b 84.5 71
5--6 130 121.25
7--8 243 155.5
11-12 424 230
13--14 533 323.5
y = 1.1658x + 155.87
y = 2.6592x - 183.51
y = 0.9055x + 20.211
Age (ka)
Depth (cm)
0 50 100 150 200 250 300 350

Caption. Figure S1. Age-depth plots for Arctic cores used in paleothermomtry. An age model for each core was developed using tiepoints for segments of the core from reference # 9 (Marzen et al. 2016). All cores used ^{14}C dates for the first age tie point, except P21, which has no ^{14}C dates. Estimates of glacial terminations determined by ostracode and foraminiferal density and local stratigraphic markers tuned to the deep-sea oxygen isotope stratigraphy (reference # 17, Lisiecki and Raymo 2005) were used for other tie points. Age models for HLY-6 and P23 back to 900 ka were taken from reference # 15 (Cronin et al. 2010) and reference # 13 (Polyak et al. 2013), ages older than 900 ka in P23 were extrapolated from the age model of Polyak et al. (2013) and are considered preliminary.

Table S1

Core	*B. aculeata* zone mid-depth (cm)	*T. egelida* zone mid-depth (cm)	pink white layer 1 mid-depth (cm)	pink white layer 2 mid-depth (cm)
P9	163.25-181.75	413.75-427.25	N/A	N/A
P40	86.5-114	342.5-403	N/A	N/A
P39	105.5-119.5	288.5-332	210.5	145.5
P30	140-186	586-624	164	418
P21	34.5-55.5	204.5-224.5	179.5	103

Table S1 Caption

The following tie points were used to correlate among the cores and develop age models

- **MIS 1- RC**
- **MIS5a- *Bulimina aculeata* benthic foraminiferal zone**

- **MIS 11- *Turborotalia egelida* plantic foraminiferal zone**
- **MIS 8--7- pink white layer 1 (when applicable)**
- **MIS 5 (early) - pink white layer 2 (when applicable)**

Additional references

9. Marzen, R., DeNinno, L. & Cronin T. M. Calcareous Microfossil-Based Orbital Cyclostratigraphy in the Arctic Ocean. *Quat. Sci. Rev.* **149**, 109-121 (2016).

17. Lisiecki, L. E. & Raymo, M. E. A Pliocene-Pleistocene stack of 57 globally distributed benthic $\delta^{18}O$ records. *Paleoceanography* **20**, doi: 10.1029/2004PA001071 (2005).

31. Cronin, T. M. et al. Quaternary ostracode and foraminiferal biostratigraphy and paleoceanography in the western Arctic Ocean. *Mar. Micropaleontol.* **111**, 118-133 (2014).

32. DeNinno, L. H., Cronin, T. M., Rodriguez-Lazaro, J. & Brenner, A. An early to mid-Pleistocene deep Arctic Ocean ostracode fauna with North Atlantic affinities. *Palaeogeography, Palaeoclimatology, Palaeoecology* **419**, 90-99 (2015).

33. Yasuhara, M., Stepanova, A., Okahashi, H., Cronin, T. M., & Brouwers, E. M. Taxonomic revision of deep-sea Ostracoda from the Arctic Ocean. *Micropaleontology* **60**, 399-444 (2014).

34. Gemery, L., et al. An Arctic and Subarctic ostracode database: biogeographic and paleoceanographic applications. *Hydrobiologia*, 1-37 (2015).

35. Cadot, H. M. & Kaesler, R. L. Magnesium content of calcite in carapaces of benthic marine Ostracoda. *Univ. Kansas Paleontological Contributions* **87**, 1-23 (1977).

36. Corrège, T. Preliminary results of paleotemperature reconstruction using the magnesium to calcium ratio of deep-sea ostracode shells from the late Quaternary of Site 822, Leg 133, (western Coral Sea). *Proceedings of the Ocean drilling Program, Scientific Results* **133**, 175-180 (1993).

37. Dwyer, G. S., Cronin, T. M., Baker, P. A., Raymo, M. E., Buzas, J. S. & Corrège T. North Atlantic deepwater temperature change during late Pliocene and late Quaternary climatic cycles. *Science* **270**, 1347–1351 (1995).
38. Dwyer, G. S., Cronin, T. M. & Baker, P. A. Trace elements in marine ostracodes, *Ostracoda: Applications in Quaternary Research* **131**, 205–225 (2002).
39. Yu, J., Broecker, W. S., Elderfield, H., Jin, Z., McManus, J., & Zhang, F. Loss of carbon from the deep sea since the Last Glacial Maximum. *Science* **330**, 1084-1087 (2010).
40. Sosdian, S. & Rosenthal, Y. Deep-sea temperature and ice volume changes across the Pliocene-Pleistocene climate transitions. *Science* **325**, 306-310 (2009).
41. Henrich R., Baumann, K. H., Huber, R., & Meggers, H. Carbonate preservation records of the past 3 Myr in the Norwegian–Greenland Sea and the northern North Atlantic: implications for the history of NADW production. *Marine Geology* **184**, 17-39 (2002).
42. Cronin, T. M., Gemery, L., Briggs Jr., W. M., Jakobsson, M., Polyak, L., & Brouwers, E. M. Quaternary Sea-ice history in the Arctic Ocean based on a new Ostracode sea-ice proxy. *Quat. Sci. Rev.* **29**, 3415-3429 (2010).
43. Karanovic, I. & Brandão, S. N. The genus *Polycope* (Polycopidae, Ostracoda) in the North Atlantic and Arctic: taxonomy, distribution, and ecology. *Systematics and Biodiversity* **14**, 2, 198-223 (2016).
44. Arndt, J. E., Niessen, F., Jokat, W. & Dorschel, B. Deep water paleo-iceberg scouring on top of Hovgaard Ridge–Arctic Ocean. *Geophys. Res. Lett.* **41**, 2014GL060267 (2014).
45. Polyak, L., Edwards, M.H., Coakley, B.J. & Jakobsson, M. Ice shelves in the Pleistocene Arctic Ocean inferred from glaciogenic deep-sea bedforms. *Nature* **410**, 453–459 (2001).
46. Polyak, L., Darby, D., Bischof, J. & Jakobsson, M. Stratigraphic constraints on late Pleistocene glacial erosion and deglaciation of the Chukchi margin, Arctic Ocean. *Quat. Res.* **67**, 234–245 (2007).
47. Dove, D., Polyak, L. & Coakley, B. Widespread, multi-source glacial erosion on the Chukchi margin, Arctic Ocean. *Quat. Sci. Rev.* **92**, 112–122 (2014).

48. Engels, J. L., Edwards, M. H., Polyak, L. & Johnson, P. D. Seafloor evidence for ice shelf flow across the Alaska–Beaufort margin of the Arctic Ocean. *Earth Surf. Processes Landforms* **32**, 1–17 (2008).
49. Niessen, F. et al. Repeated Pleistocene glaciation of the East Siberian continental margin. *Nat. Geosci.* **6**, 842–846 (2013).
50. Jakobsson, M. et al. An Arctic Ocean ice shelf during MIS 6 constrained by new geophysical and geological data. *Quat. Sci. Rev*. **29**, 3505–3517 (2010).
51. Jakobsson, M., et. al. Arctic Ocean glacial history. *Quat. Sci. Rev.* **92**, 40-67, doi: 10.1016/j.quascirev.2013.07.033 (2014).
52. Stokes, C. R., et al. On the reconstruction of palaeo-ice sheets: recent advances and future challenges. *Quat. Sci. Rev.* **125**, 15-49 (2015).
53. Elderfield, H., et al. Evolution of ocean temperature and ice volume through the mid-Pleistocene climate transition. *Science* **337**, 704-709 (2012).
54. Rohling, E. J., et al. Antarctic temperature and global sea level closely coupled over the past five glacial cycles. *Nature Geosciences* **2**, 500-504 (2009).
55. Shakun, J. D., et al. An 800-kyr record of global surface ocean $\delta^{18}O$ and implications for ice volume-temperature coupling. *Earth and Planetary Science Letters* **426**, 58-68 (2015).
56. Lüthi, D., et al. High-resolution carbon dioxide concentration record 650,000–800,000 years before present. *Nature* **453,** 379-382 (2008).
57. Hao, Q., et al. Delayed build-up of Arctic ice sheets during 400,000-year minima in insolation variability. *Nature* **490**, 393-396 (2012).
